# A study for precision diagnosing and treatment strategies in difficult-to-treat AIDS cases and HIV-infected patients with highly fatal or highly disabling opportunistic infections: Study protocol for the optimal early intervention for cryptococcal antigenemia in HIV-infected patients

**DOI:** 10.1097/MD.0000000000022874

**Published:** 2020-10-30

**Authors:** Feng Sun, Xiao-Lei Xu, Yan-Qiu Lu, Yao-Kai Chen

**Affiliations:** Division of Infectious Diseases, Chongqing Public Health Medical Center, Chongqing, China.

**Keywords:** cryptococcal antigenemia, early intervention, fluconazole, HIV, opportunistic infections

## Abstract

**Background:**

Asymptomatic cryptococcal antigenemia is a state of cryptococcal infection commonly seen in immunocompromised HIV-infected persons. Without early intervention, a proportion of HIV-infected persons with cryptococcal antigenemia may go on to develop cryptococcosis, especially cryptococcal meningitis, which is associated with high mortality. The benefits of antifungal intervention and optimal therapeutic intervention regimens for HIV-infected persons with cryptococcal antigenemia remain controversial. We therefore designed the present study in order to investigate the necessity of, and the optimal regimens for antifungal intervention in the clinical management of cryptococcal antigenemia in HIV-infected populations.

**Methods/Design:**

This study will be an open-labeled, multi-center, prospective, randomized controlled trial, and 450 eligible participants will be randomized into a control arm and 2 intervention arms at a 1:1:1 ratio, with 150 subjects in each arm. Participants in the control arm will not receive antifungal treatment during the study period. Participants in intervention arm 1 will receive oral fluconazole 800 mg/day for 2 weeks, followed by 400 mg/day for 8 weeks and 200 mg/day for 42 weeks, and participants in intervention arm 2 will receive oral fluconazole 400 mg/day for 52 weeks. The primary outcome is the incidence of CM among the 3 groups during the study period. The secondary outcomes include the differences in all-cause mortality, proportion of patients reverting to blood CrAg negativity, change of CrAg titers, and adverse events among the 3 groups during the follow-up period.

**Discussion:**

We envisage that the results of this study will reveal the necessity of, and the optimal therapeutic regimens for, antifungal intervention in clinical management of HIV-infected patients with cryptococcal antigenemia.

**Trial registration:**

The study was registered as one of the 12 clinical trials under a general project at the Chinese Clinical Trial Registry on February 1, 2019, and the registration number of the general project is ChiCTR1900021195.

## 1 Introduction

Cryptococcal antigenemia refers to a primary infective state, in which cryptococcal antigen can be detected in the blood only, while the infected patient lacks clinical or other evidence of cryptococcosis, including symptoms, signs, laboratory abnormalities, or imaging abnormalities. Cryptococcal antigenemia often occurs in HIV-infected patients with CD4 + T-cell counts <100 cells /μl, but may occur in HIV-infected patients with CD4+ T-cell counts between 100 cells /μl and 200 cells/μl, and even some patients with CD4 + T-cell counts >200 cells /μl may be serologically positive for cryptococcal antigen (CrAg).^[1]^ In the absence of active intervention, asymptomatic cryptococcal antigenemia may develop cryptococcosis, predominantly cryptococcal meningitis (CM), which is the most common clinical form of cryptococcosis, and causes 625 000 annual deaths globally.^[2]^ There are a few studies in favor of early intervention with fluconazole in order to improve survival in HIV-infected patients with cryptococcal antigenemia. One prospective cohort study, for example, demonstrated that fluconazole (200–400 mg/day) intervention for cryptococcal antigenemia is likely to improve long-term survival.^[3]^

However, a few studies failed to observe survival benefit for pre-emptive fluconazole treatment among HIV-infected patients with CrAg positivity.^[4]^ The aforementioned studies show that the role of preemptive treatment with fluconazole in HIV-infected patients with cryptococcal antigenemia remains controversial. In the present study, we will set up a control group, to specifically clarify whether early intervention with fluconazole is therapeutically beneficial for patients with cryptococcal antigenemia.

The 2011 WHO guidelines recommend the regimen of fluconazole 800 mg/day for 2 weeks, then 400 mg/day for 8 weeks, and continued maintenance with fluconazole 200 mg/day for isolated serum CrAg positivity patients with CD4 + T-cell counts <100 cells /μl.^[5]^ While in the newest guidelines of the National Institutes of Health (NIH), for this group of patients, treatment with fluconazole (400 mg daily for 12 months) is appropriate.^[6]^ To date, there are no relevant studies comparing these 2 interventions. We therefore designed 2 specific therapeutic intervention regimens, in accordance with WHO recommendations and NIH guidelines, in order to explore the optimal regimen for the treatment of HIV-infected patients with cryptococcal antigenemia.

## 2 Methods/Design

### 2.1 Research objective

This study aims to investigate the optimal early intervention for cryptococcal antigenemia in HIV-infected patients.

### 2.2 Study design

**Figure 1:**
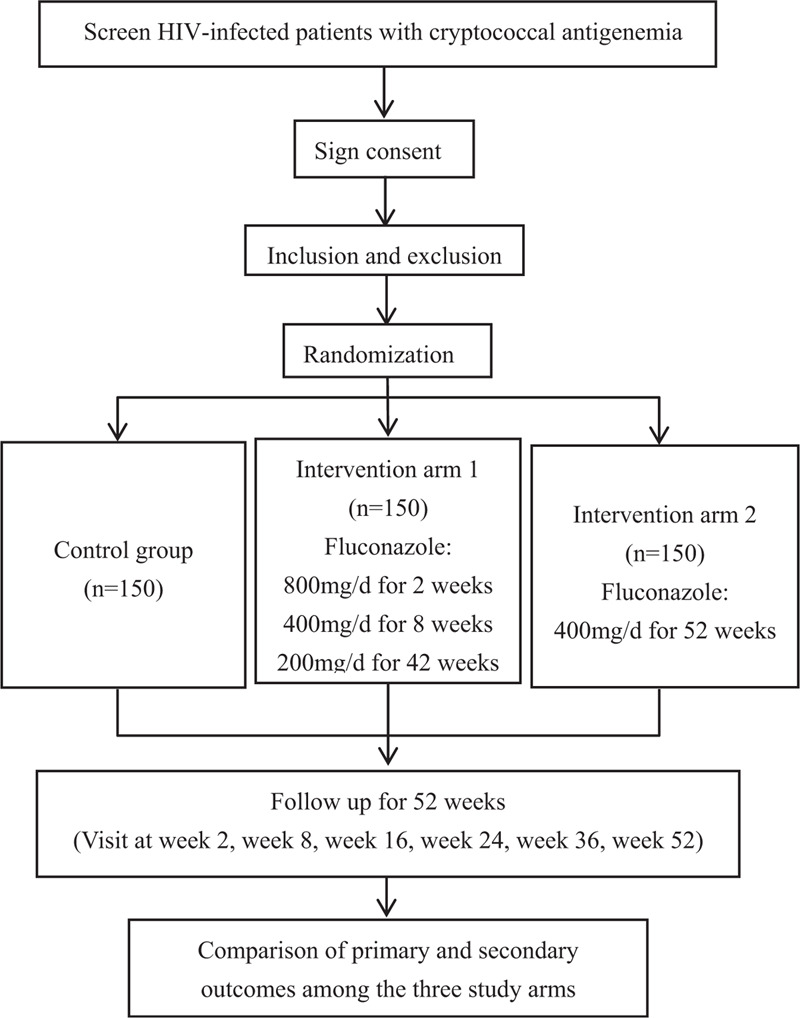
Flow chart of study design.

This study will be conducted as an open-labeled, multi-center, prospective, randomized, controlled trial. Four hundred fifty subjects will be recruited from 17 hospitals including Chongqing Public Health Medical Center, Capital Medical University Beijing You’an Hospital, the Forth Affiliated Hospital of Harbin Medical University, the Second People's Hospital of Tianjin, the First Hospital of Changsha, the Eighth People's Hospital of Guangzhou, Liuzhou General Hospital, the Third People's Hospital of Guilin, the Third People's Hospital of Shenzhen, Guiyang Public Health Clinical Center, Public Health Clinical Center of Chengdu, Kunming Third People's Hospital, Yunnan Provincial Infectious Disease Hospital, the Fourth People's Hospital of Nanning, Guangxi Longtan Hospital, the First Affiliated Hospital of Zhejiang University, and Xixi Hospital of Hangzhou for this study. This protocol was designed and written in strict accordance with the Standard Protocol Items: Recommendations for Interventional Trials (SPIRIT) statement.^[7]^ The procedures for subject enrolment, drug intervention and clinical assessment are described in Figure 1. After confirming cryptococcal antigenemia and signing informed consent, all subjects in the study will attend 9 scheduled follow-up visits for 52-weeks. Follow-up visits will be arranged for subjects as scheduled in Table 1. Blood, urine and cerebrospinal fluid samples (CSF) will be collected for laboratory testing, including for routine blood examination, liver function, renal function, routine urinalysis, routine CSF analysis, CSF biochemistry, electrolytes, myocardial enzymes, blood amylase, urine human chorionic gonadotropin (HCG), CD4+/CD8+ ratio, serum CrAg (qualitative and quantitative), Cryptococcus culture, CSF CrAg, CSF India ink staining, and HIV-1 RNA level. All items considered and tested for during the follow-up period are listed in Table 1.

**Table 1:**
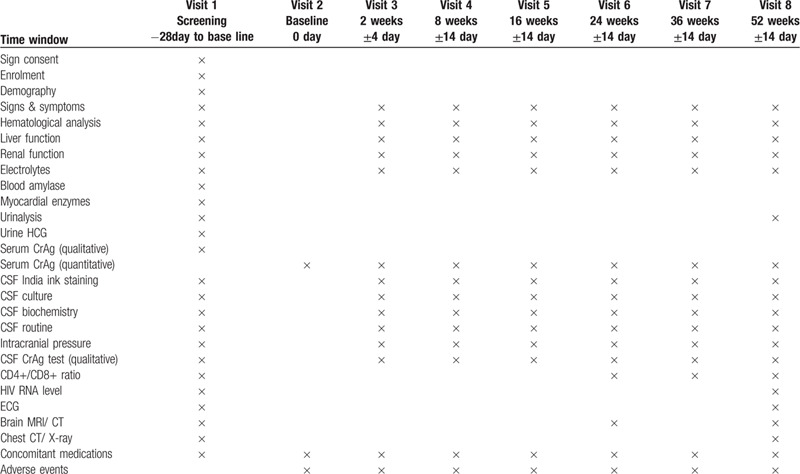
Study time points for enrolment, intervention and assessment.

## 3 Participants

### 3.1 Diagnostic criteria

The diagnostic criteria of HIV infection and AIDS in this study are consistent with the DHHS Guidelines for the Prevention and Treatment of Opportunistic Infections in HIV-Infected Adults and Adolescents.^[6]^

The diagnosis of cryptococcal antigenemia in HIV-infected persons will have to meet the following criteria:

1. Blood cryptococcal antigen test positive (titer >1:8) by latex agglutination test (LA);^[8]^
2. Absence of abnormities on CSF examination;
3. Absence abnormities on lung and brain imaging;
4. Absence of any clinical signs or symptoms of cryptococcosis.

### 3.2 Inclusion criteria

Confirmed HIV-infected persons meeting the following criteria will be included in our study:

1. Aged 18 years or over;
2. Meeting the diagnostic criteria of cryptococcal antigenemia;
3. CD4 T-cell count <200 cells/μL;
4. Voluntarily sign informed consent, and are willing to be followed-up;
5. The overall condition of the subject does not affect the evaluation and completion of the trial.

### 3.3 Exclusion criteria

Subjects will be excluded from the study if they satisfy the following criteria:

1. Are allergic or intolerant to fluconazole;
2. Cryptococcal antigen titers ≥1: 512;^[9]^
3. Have hemoglobin (Hb) <60 g/L, white blood cell count (WBC) <1.0 × 10^9/L, neutrophil count (N) <0.5 × 10^9/L, platelet count (PLT) <50 × 10^9/L, blood amylase (AMS) >2 × UNL, serum creatinine (Scr) >1.5 × UNL, aspartate aminotransferase (AST) /alanine aminotransferase (ALT) /alkaline phosphatase (ALP) >5 times of UNL, total bilirubin (TB) >2 × UNL, serum creatine phosphokinase (CK) >2 × UNL;
4. Previously diagnosed or currently diagnosed with cryptococcosis;
5. Are using antifungals;
6. Have other co-morbid diseases that may affect the efficacy and execution of this study, including but not limited to heart disease, brain disease, lung disease, kidney disease, neoplastic disease, and other systemic diseases;
7. Are pregnant or lactating women;
8. Are intravenous drug users;
9. Are not of Chinese nationality.

### 3.4 Randomization

A random number sequence will be generated for each subject through Medical Research Platform (http://www.51yyt.org/FrontPage/login.aspx?Inviter=) after obtaining written informed consent. Once eligibility is confirmed, subjects will immediately be randomized into the control arm, intervention arm 1, or intervention arm 2, at a 1:1:1 ratio.

### 3.5 Data collection and quality assurance

Data collection will begin at baseline, and will continue during all scheduled visits defined in the study protocol. All data including adverse events (AEs) in this study will be documented on case report forms (CRFs), and subsequently will be input into the trial database at Medical Research Platform (http://www.51yyt.org/FrontPage/login.aspx?Inviter=).

In order to ensure data quality and integrity, all investigators will be trained strictly in accordance with a Standard Operating Procedure (SOP) trial manual, and startup meetings will be held for all participating hospitals. The investigators will review CRFs to ensure that all included data is in agreement with original medical records. Missing values will be checked, and significantly abnormal data, or data that are outside the clinically acceptable range (exceeding 20% of the normal value) will be required to be explained by the attending physician. Drop-outs and adverse events at any period will also be recorded in time.

### 3.6 Intervention

#### 3.6.1 Antifungal therapy

Patients randomized to intervention arm 1 will receive fluconazole therapy orally at a prescribed dose of 800 mg/day for 2 weeks, followed by 400 mg/day for 8 weeks, and then 200 mg/day for 42 weeks, and those randomized to intervention arm 2 will receive oral fluconazole at a dose of 400 mg/day for 52 weeks.

#### 3.6.2 ART therapy

All subjects will receive antiretroviral therapy. The preferred antiretroviral regimen will be TDF (300 mg/d) +3TC (300 mg/d) +EFV (600 mg/d), and other regimens are optional. The specific ART regimens prescribed to each patient will be selected by attending doctors, and determined by a synthesis of available clinical and other medical parameters of individual patients. The timing for ART initiation for ART-naïve patients will be 5 weeks after confirmation of cryptococcal antigenemia. For those patients on ART, their regimens may continue, or may be switched to a new regimen, subject to the judgment of attending doctors. Those in the control group will not receive fluconazole therapy.

Individuals that are randomized into the control group will undergo strict supervision, and will be watched closely. If patients in the control arm develop any potential clinical signs that may be indicative of meningitis, viz. low-grade fever, headaches, nausea, vomiting, an altered mental state, confusion, irritability, memory disorders, behavioral changes, drowsiness, neck stiffness, visual disturbances, increased cerebral pressure, or are found to have increased CSF protein, mild or moderate serum or CSF leukocyte elevation, reduced CSF glucose, display Cryptococcus in CSF by India ink staining, become CSF CrAg-positive, or become CSF Cryptococcus culture positive, immediate therapeutic antifungal drugs for CM will be initiated.

### 3.7 Endpoints

1. Clinical diagnosis of CM or any other symptomatic cryptococcosis
2. Cryptococcal antigen titers≥1: 512 on 2 consecutive readings;^[9]^
3. CD4 cell counts increase beyond ≥200 cells/μl in 2 consecutive tests 3 months apart, and blood or CSF CrAg results are negative in 2 consecutive tests 3 months apart;
4. Completion of the entire medication and follow-up cycle in accordance with the trial protocol;
5. Severe adverse reactions or drug intolerance during the study period.

### 3.8 Outcomes

#### 3.8.1 Primary outcome

Comparison of the incidence of CM among the 3 groups during the study period.

### 3.9 Secondary outcomes

Comparison of the differences in all-cause mortality, proportion of patients reverting to blood CrAg negativity, change of CrAg titers, and adverse events among the 3 groups during the follow-up period.

### 3.10 Sample size

This trial is a randomized, controlled trial with 3 parallel groups, without blinding, and with a 1:1:1 allocation ratio. The sample size will be 150 subjects per arm, based on a power of 80%, with a level of confidence of 95%. We expect the drop-out rate to be approximately 20%.

### 3.11 Statistical analysis

Primary and secondary outcome analysis will be conducted using the Intent-to-Treat Exposed (ITT-E), and the per-protocol (PP) analysis set. The ITT-E analysis set consists of all randomized subjects, whether they have attended the entire follow-up period, or not. The PP analysis set excludes patients who do not follow the study protocol. The last-observation-carried-forward (LOCF) method will be used to ensure the accuracy of conclusions if any subject withdraws from the study. Primary and secondary outcomes will be compared among the 3 groups using time-to-event methods with Cox proportional-hazards models. Categorical variables will be reported as number and percentage, and compared using Fishers exact test, or the Chi-Squared test, and continuous variables will be reported as mean with standard deviation (SD), or median with interquartile range (IQR), and compared via the analysis of variance (ANOVA), and the Kruskal–Wallis non-parametric test. A *P* value of <.05 will be deemed to confer statistical significance. All statistical analyses will be performed using Statistical Package for the Social Sciences (SPSS) software, Version 24 (IBM SPSS Inc., Chicago, IL, USA).

### 3.12 Ethics and dissemination

This study has been approved by The Ethics Committee of Chongqing Public Health Medical Center (2019-003-02-KY), and has been registered as one of the 12 trials under the name of a general project at clinicaltrials.gov under registration number ChiCTR1900021195. The approval of the Ethics Committees for each participating site will be secured before initiation of patient recruitment. We will share the results through a published medical journal article, or a conference presentation after completion of the study.

## 4 Discussion

A limited number of past research have investigated the treatment strategy of cryptococcal antigemenia in HIV-infected persons; however, the outcomes of most previous studies have low power due to limited sample size, and lack exploration of optimal fluconazole therapeutic doses in this population.^[3,8]^ In addition, most contemporary data in the literature exploring fluconazole treatment for cryptococcal antigenemia and CM are from African, European, or South-east Asian countries, and there have been no appropriate large-scale cohort studies conducted in this population in China as yet.^[10–12]^ Our large cohort study will be superior to previous studies in terms of sample size and power; hence the results and outcomes of our investigation will tend to be more compelling. Unlike similar past studies, our study utilizes standardized recommended treatment regimens in accordance with contemporary international guidelines. Thus, the present study will help explore the possibility of an optimal therapeutic intervention regimen for patients with cryptococcal antigenemia.

We expect that there will be a few challenges during the implementation of our study. One potential concern is that the overall incidence of cryptococcal antigenemia in the HIV-infected patient population is gradually decreasing due to the widespread use of highly effective modern ART. It is therefore possible that we may not achieve our target number of subjects in each intervention arm in the scheduled time as set out in this protocol. In addition, the worrying adverse reaction profile of fluconazole, and its consequent effect on patient compliance, and the long prescribed 52-week follow-up period, may be challenging for some patients, and thus the patient drop-out rate may be higher than that anticipated in this protocol. In order to overcome these challenges, and to ensure the consistent, highest-quality implementation of this study, we will: a) conduct a multi-center trial, and recruit participants from 17 large designated hospitals for HIV care; b) educate participants with regards to the aims and outcomes of our study as comprehensively as possible in order to enhance the likelihood of their prolonged adherence to our study protocol; and, c) provide a comprehensive consulting service to participants during the study period, which will extend into the period after study completion. It is hoped that our perseverance, and our patient cohorts fortitude during the implementation of this study, will have eventual positive outcomes for HIV-positive patients afflicted with cryptococcal antigenemia around the world.

## Acknowledgments

We appreciate the assistance of Dr. Vijay Harypursat from New Zealand for language improvement and revision of the manuscript.

## Author contributions

Xiao-lei Xu and Yan-qiu Lu conceived the study. Feng Sun designed the study protocol. Feng Sun drafted the manuscript. Xiao-lei Xu developed the first draft of the manuscript. Yan-qiu Lu registered the study. Yao-kai Chen sought funding and ethical approval. All authors read and approved the manuscript.

**Conceptualization:** Feng Sun, Xiao-lei Xu, Yan-qiu Lu.

**Funding acquisition:** Yao-kai Chen.

**Methodology:** Feng Sun.

**Project administration:** Xiao-lei Xu, Yan-qiu Lu.

**Resources:** Yao-kai Chen.

**Writing – original draft:** Feng Sun.

**Writing – review & editing:** Yao-kai Chen.
